# *Gasterophilus intestinalis* infestation in lion (*Panthera leo*) and plains zebra (*Equus quagga*) in the Serengeti ecosystem: Morphological and molecular profiling[Fn FN1]

**DOI:** 10.1051/parasite/2024060

**Published:** 2024-09-27

**Authors:** Barakaeli Abdieli Ndossi, Eblate Ernest Mjingo, Maulid Mzinga Mdaki, Marry Wokusima Zebedayo, Seongjun Choe, Mohammed Mebarek Bia, Heejae Yang, Sungbo Seo, Keeseon S. Eom

**Affiliations:** 1 Tanzania Wildlife Research Institute P.O. Box 661 206 Njiro Road 2113 Lemara Arusha Tanzania; 2 Department of Parasitology, Parasitology Research Center and International Parasite Resource Bank, Chungbuk National University, School of Medicine Cheongju 28644 Republic of Korea; 3 Cocoon Inc. #704, 194-41, Osongsaengmyeong 1-ro, 116, Uiryodanji-gil, Osong-eup, Heungdeok-gu Cheongju-si Chungcheongbuk-do 28161 Republic of Korea

**Keywords:** *Gasterophilus intestinalis*, Serengeti ecosystem, Maswa, Lion, Zebra Tanzania

## Abstract

This study was conducted to clarify the host specificity and the geographical distribution of *Gasterophilus* species (Diptera, Oestridae) in the Serengeti ecosystem. A total of 317 larvae were recovered from two common zebras (*Equus quagga*, formerly *Equus burchellii*) in Maswa Game Reserve, and 58 larvae were recovered from an African lion (*Panthera leo*) in the Serengeti National Park. The study emphasizes the rare occurrence of *Gasterophilus* sp. in lions, shedding light on the broader life cycle and physiological implications for hosts. Genetic analysis of *cox*2 genes from *Gasterophilus* species, sourced from a single geographic location, reveals significant genetic distinctions and host specificity. This study reports the first case of *G. intestinalis* infestation in an African lion in the Serengeti ecosystem, extending its known range from zebras and other equids, and highlighting ecological and veterinary implications. This unusual prey-predator transmission highlights the value of molecular taxonomic tools in clarifying host-parasite dynamics and guiding targeted conservation strategies.

## Abstract (fr)

Cette étude a été menée pour clarifier la spécificité de l’hôte et la répartition géographique des espèces de *Gasterophilus* (Diptera, Oestridae) dans l’écosystème du Serengeti. Au total, 317 larves ont été récoltées chez deux zèbres communs (*Equus quagga*, anciennement *Equus burchellii*) dans la réserve de gibier de Maswa, et 58 larves ont été récoltées chez un lion d’Afrique (*Panthera leo*) dans le parc national du Serengeti. L’étude souligne la rareté de l’occurrence de *Gasterophilus* sp. chez les lions, mettant en lumière le cycle biologique plus large et les implications physiologiques pour les hôtes. L’analyse génétique des gènes *cox2* des espèces de *Gasterophilus*, provenant d’un seul lieu géographique, révèle des distinctions génétiques et une spécificité d’hôte significatives. Cette étude rapporte le premier cas d’infestation par *G. intestinalis* chez un lion africain dans l’écosystème du Serengeti, étendant son aire de répartition déjà connue chez les zèbres et autres équidés, et mettant en évidence des implications écologiques et vétérinaires. Cette transmission inhabituelle de proie à prédateur souligne l’intérêt des outils de taxonomie moléculaire pour clarifier la dynamique hôte-parasite et guider les stratégies de conservation ciblées.

## Introduction

Genus *Gasterophilus* Leach (Diptera: Oestridae, Gasterophilinae) consists of obligatory parasites that affect both domestic and wild equids [2, 12, 32]. Prevalent species such as *G. intestinalis* De Geer, 1776, *G. nasalis* Linnaeus, 1758, and *G. haemorrhoidalis* Linnaeus, 1758 have been reported consistently worldwide [47]. Additionally, *G. flavipes* Oliver, 1811, *G. inermis* Brauer, 1858, *G. meridionalis* Pillers & Evans, 1926, *G. nigricornis* Loew, 1863, *G. pecorum* Fabricius, 1794 and *G. ternicinctus* Gedoelst, 1912 are identified in the Palearctic and Afrotropical Regions [13, 37].

Horses, donkeys and Burchell’s zebra are frequently infected by the larval stages of genus *Gasterophilus*, with sporadic cases noted in cows, goats, sheep, wild ass, lion and rhinoceros [47]. Female *Gasterophilus* lay 150–1000 eggs on the host’s hair, with hatching influenced by temperature and humidity during grooming [27]. After ingestion, the larvae moult in the mouth, progressing to the second stage before moving into the stomach, where second and third stage larvae attach to the stomach lining near the oesophageal-cardiac junction [3].

Severe infestation characterised by clinical and pathological features such as oesophageal paralysis, peritonitis, squamous cell tumours, chronic gastritis, ulcerated stomach, stomach rupture, anaemia and potential fatality can result from a high burden in the host [5, 8, 16]. Instances of external ophthalmomyiasis induced by *Gasterophilus* species larvae have been documented in humans [26].

The identification of *Gasterophilus* species is linked to the host-parasite relationship, influencing their ecological adaptation and biodiversity [31]. Despite the rich biodiversity of domestic and wild animals in Tanzania, limited information on *Gasterophilus* infestation has been documented, calling for further exploration of their host-specificity and their geographical distribution [21]. Intraspecific variation with *Gasterophilus* species has been observed [24, 47]. However, clear descriptions of their larval morphology based on microscopy observations and molecular techniques remain elusive [8, 23].

The importance of reporting these arthropods extends beyond scientific findings, offering valuable taxonomic insight into the biogeographical distribution of *Gasterophilus* species. This study marks the initial documentation of *Gasterophilus* infestation in African lion and zebra within the Great Serengeti ecosystem in Tanzania. The clarification of the larval stage involves a rigorous examination that integrates both morphological and molecular identification methodologies, contributing valuable biological and scientific insights for researchers in this field. The use of mitochondrial genes suggests their potential as molecular markers for taxonomic differentiation and evolutionary studies of insects [31, 32].

## Materials and methods

### Ethics statement

The objectives and procedures of this study, which is part of an ongoing project, were reviewed and approved by the Joint Management Research Committee of the Tanzania Wildlife Research Institute. Additionally, research permits were granted by the Commission for Science and Technology Tanzania (COSTECH-2023-878-ER-2021-265). Field collections of third-instar larvae were conducted in the Serengeti ecosystem, following the necessary permissions to enter these protected areas. All larvae were isolated exclusively from naturally dead wild animals encountered during the survey.

### Study area

This study was conducted in Maswa Game Reserve and Serengeti National Park along the Serengeti ecosystem in Tanzania between 2015 and 2023 (Fig. 7). A total of 375 third instar larvae (L3) of *G*. *intestinalis* were recovered from the gastrointestinal tracts (stomach, duodenum) of three host animals. Specifically, 317 larvae were recovered from two common zebra (*Equus quagga*, formerly *Equus burchellii*) in Maswa Game Reserve, and 58 larvae were recovered from an African lion (*Panthera leo*) in the Serengeti National Park. The specimens were fixed with some flesh to retain original morphological features.

### Morphological preparation

Morphology features were studied by using an Olympus SZX16 stereoscopic microscope (Olympus Corp., Tokyo, Japan). Microscopic observation was conducted based on Zumpt [47]. Three larvae of each species from zebra and African lion were selected for scanning electron microscopy (SEM). The larvae were trimmed and fixed to 2.5% glutaraldehyde (0.1 M phosphate buffer, pH 7.2 ~ 7.4) overnight. The specimen was then washed with 0.1 M phosphate buffer 15 ~ 20 min 3 times and transferred to 30, 50, 60, 70, 80, 90, 95, 100 and 100% ethanol (each step 30 min). The dried specimen was then transferred to isoamyl acetate or hexamethyldisilazane twice (each step 30 min) and then dried before being taken to the scanning electron microscope (SEM) for observation. After gold coating, specimens were observed by SEM (Hitachi S-570, Tokyo, Japan). The samples were mounted on stubs with double-sided adhesive tape and left in a desiccator overnight to dry thoroughly, then coated with gold and examined using a Hitachi S-570 SEM (Hitachi Co. Tokyo, Japan). Morphological identifications were conducted based on Principato and Tosti [34].

### PCR and DNA sequencing

Prior to DNA extraction, the larvae were picked from the collected flesh and homogenised by washing extensively in phosphate-buffered saline overnight in the shaker (Laboshaker R100 Gyrozen^®^ Yuseong-gu, Daejeon 305-301, Korea) for 24 h. After washing, the larvae were chopped (25 mg) and homogenised in animal tissue lysis buffer and proteinase K in a 1.5 mL microcentrifuge tube and allowed to melt into suspension overnight at 56 °C. The incubated larvae sample was extracted using a QIAamp DNA mini-Kit, following the manufacturer’s procedures (QIAGEN, Valencia, CA, USA). Genomic DNA was dissolved in 50 μL of TE buffer (10 mM Tris/1 mM EDTA).

The mitochondrial *Cox* 2 fragment was amplified by primers GASF (5′-ATG GCAGAT TAG TGC AAT GG-3′) and GASR (5′-GTTTAA GAG ACC AGT ACT TG-3′) [38]. The PCR amplification was performed using 50 ng of genomic DNA template in 25 μL reaction mixtures consisting of 2.5 μL of 10× buffer, 12.25 μL of 2× buffer (MgCl_2_, dNTP) and 1.25 units *Taq* polymerase (TAKARA BIO INC^®^, Kusatsu, Shiga, Japan). The Genomic DNA was initially denaturised at 95 °C for 2 min followed by 30 cycles of denaturation at 95 °C for 60 s, annealing at 55 °C for 60 s, and extension at 72 °C for 60 s, with a final extension at 72 °C for 7 min. The amplification products were separated by electrophoresis in 1.5% agarose gel.

### DNA sequence analyses

DNA sequences of the mitochondrial *Cox2* were assembled using Geneious R9.1 (Biometer, Auckland, New Zealand) [19]. These sequences were compared with the published *Cox*2 genes sequences from GenBank; NC 042779/*G. haemorrhoidalis*, NC 042780/*G. inermis*, NC 042781/*G. nasalis*, KU236026/*G. intestinalis,* NC 029812/*G. pecorum*, NC 013932*/Hypoderma lineatum*, NC019640*/Rutilia goerlingiana*, HQ322500*/Exorista sorbillans*, KM881633*/Sarcophaga Africa*, and NC 024855*/Musca domestica*. Phylogenetic analysis was evaluated by employing Bayesian inference (BI) and maximum-likelihood (ML) using the partial sequences of *cox* 2 in Molecular Evolution Genetics Analysis (MEGA) software version 7.0 [22]. The HKY + G substitution model was used for sampling of *Cox2*. BI analyses were used in Bayesian Evolutionary Analysis Sampling Trees (BEAST), program version 1.10.4 [43]. The HKY substitution model sampling was chosen according to the MEGA. The nodes were assessed by bootstrapping with 1000 pseudoreplicates.

## Results

**Figure 1:**
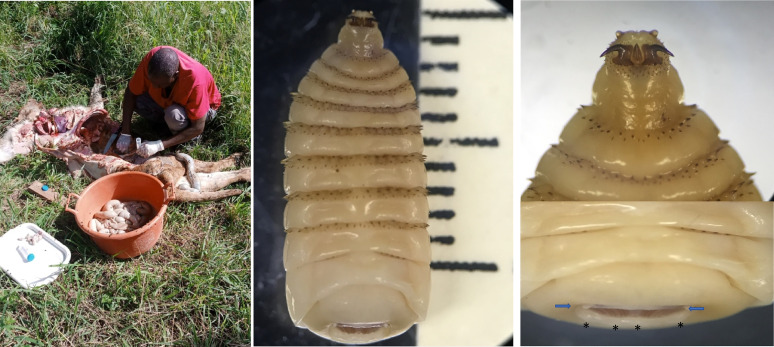
The isolation of third larval stage *Gasterophilus intestinalis* from the African lion, showing the ventral and dorsal surface of the larvae.

**Figure 2:**
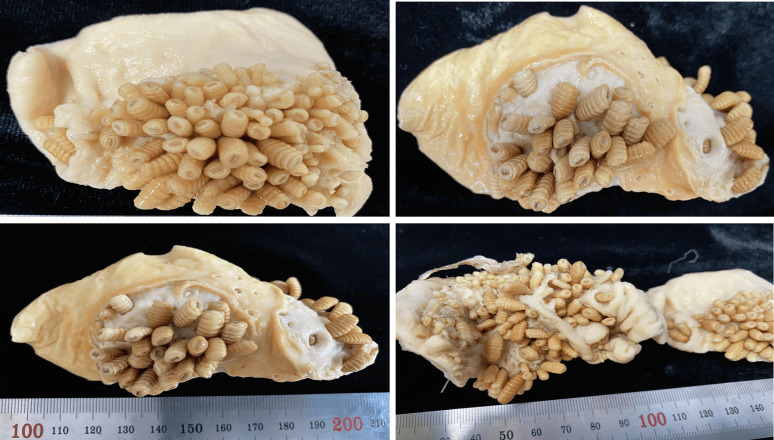
The bulk of *Gasterophilus intestinalis* attached to the gastrointestinal tissue of a zebra.

A third larval stage of *G. intestinalis* was recovered from the gastrointestinal tract of two common zebra (*n* = 317) and an African lion (*n* = 58) (Fig. 1). Key characteristics of *G. intestinalis* identification included observation of the cephalic and terminal abdominal segments of the third instar. These larvae were isolated without detachment into the tissue of the zebra’s gastrointestinal tract, and exhibited a random arrangement with mouth hooks attached to the host’s gastrointestinal tissue, resulting in crater-like lesions (Fig. 2). Morphological observations revealed the cuticular features, such as spine distribution and structure of maxillae and mandibles.

**Figure 3:**
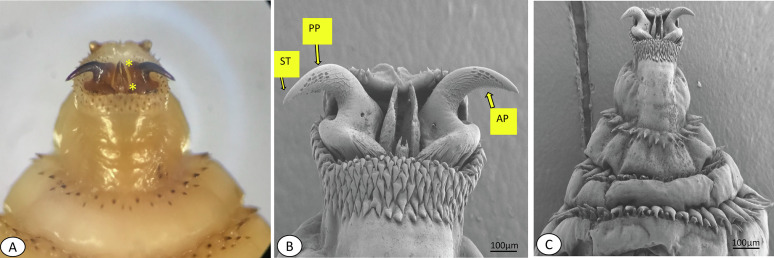
Ventral surface of thoracic and abdominal segments. Microscopic observation (A) and SEM (B). The maxillae (mouth hooks) of third instar *Gasterophilus intestinalis.* The mouth hook of the third stage showing polygonal plates (PP), angled plates (AP), and shield tip (ST). Dorsal portion of each mandible is extended into serrated lobe (Asterisk *).

**Figure 4:**
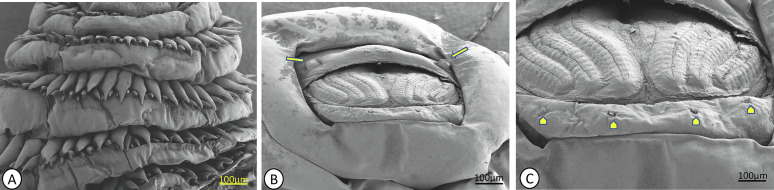
Scanning Electron Microscope (SEM) showing the arrangement of thoracic spines and the middle in (A); Opened terminal abdominal segments of the third instar *Gasterophilus intestinalis* larva; two lobes bearing sensilla (yellow arrows) (B). Four individual sensilla (arrow head) in (C).

*Gasterophilus* larvae have a cylindrical body with segmented rows of unequal spines on their dorsal and ventral surfaces, organised in two or three rows, excluding the first and last two abdominal segments. In the third larval stage, maxillae are mainly for attachment to the gastrointestinal tract during feeding on the host tissue. On the cephalic portion, there are sensory arrays on the surface of maxilla. The shape of maxillary is bent dorsally, and on the surface, there is a peg-like structure; ovoid pits lined with cuticular-pile on the dorsal and ventral surface; the linear ridges submerged and extend from the base to the tip on the posterior surface (Fig. 3). Also, the polygonal plates, angled plates, shallow pits and shield tip were observed on the cephalic portion of the third larvae of *G*. *intestinalis* (Fig. 3). The mandible is well developed dorsally extending into a notched lobe. There are two rows of unequal spines between the thoracic and anterior abdominal segments, terminating sharply to each apex. Along the posterior end of the larvae there is a wart. A pair of spiracular plates is observed enclosed by the jointed protrusion of dorsal and ventral lips along the hallow depressed cuticle at the last posterior end (Fig. 4).

### Molecular identification of *Gasterophilus intestinalis* and phylogenetic analysis

**Figure 5:**
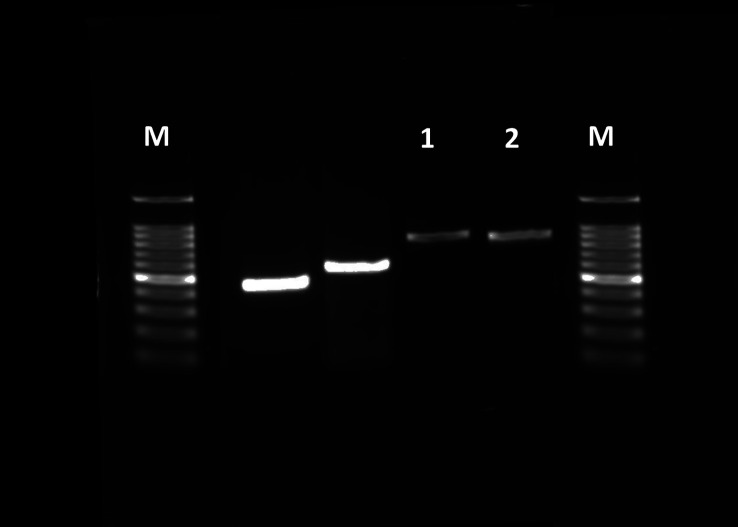
Agarose gel (1.5%) showing amplification of the *cox2* gene of *Gasterophilus intestinalis* isolates using genomic DNA of the third larvae stage of *G. intestinalis* isolated from the African lion with product size 669 bp (1) and zebra with product size 672 bp (2); negative control (3) and DNA marker (1 kb = M).

The successful amplification of cytochrome c oxidase 2 (*Cox2*) genes was verified by the observation of specific length bands on a 1.5% agarose gel (Fig. 5). Species-specific primers facilitated the amplification of approximately 669 bp for the African lion (Accession No. PP171666) and 672 bp for the zebra (Accession No. PP024641) and all the resulting partial *Cox2* gene sequences were deposited in GenBank. The taxonomic analysis utilised *Cox2* sequences from *G. intestinalis*, *G. nasalis*, and *G*. *haemorrhoidalis*, and nine additional sequences with an average length of 675 bp as reference sequences for phylogenetic assessments. In this study, pairwise distance analysis of *G*. *intestinalis* isolates from African lion and zebra exhibited 99.8% similarity, contrasting with other *Gasterophilus* species that demonstrated pairwise distances ranging from 90.9% to 84.9%.

The *Cox2* sequences from Zebra and African lion species displayed nucleotide composition frequencies, with Zebra showing A = 34.4%, C = 18.8%, G = 13.5%, T = 33.3%, and African lion featuring A = 33.6%, C = 19.3%, G = 14.4%, and T = 32.6%. The average nucleotide values for GC content were 0.32, and AT content was 0.67 in Zebra, while in lion, they were 0.33 for GC and 0.66 for AT.

**Figure 6:**
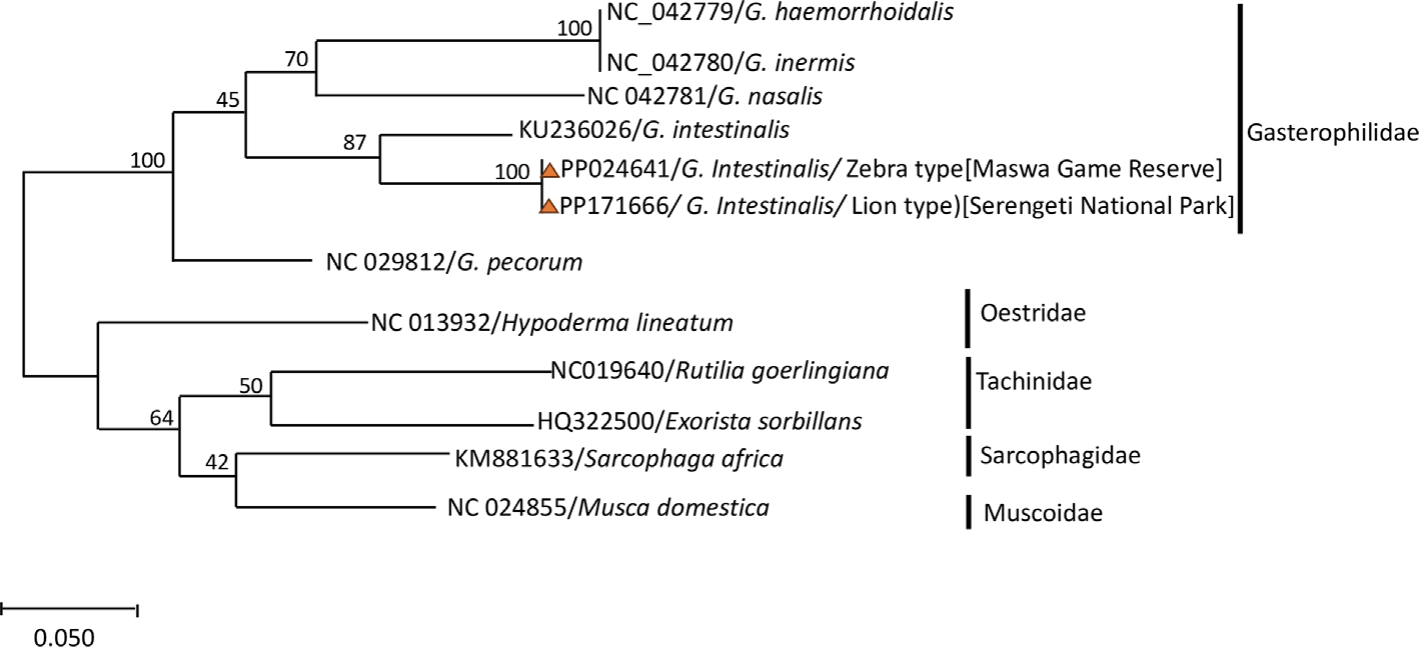
Phylogenetic tree based on mitochondrial *cox2* for *Gasterophilus intestinalis* isolates from African lion and zebra in Tanzania.

In the phylogenetic analysis, *Gasterophilus* species were classified into clades (Fig. 6). Clade I comprised Gasterophilidae, including G. *haemorrhoidalis* (NC_042779), *G. inermis* (NC_042780), *G. nasalis* (NC_042781), *G. intestinalis* (KU236026) and the current isolates from zebra (PP024641) and African lion (PP171666) in this study. Clade II demonstrated a related group with *Gasterophilus*, belonging to Oestridae, where *Hypoderma lineatum* followed by Tachinidae and Sarcophagidae, while Muscoidea was used as the outgroup in this study (Fig. 6).

**Figure 7:**
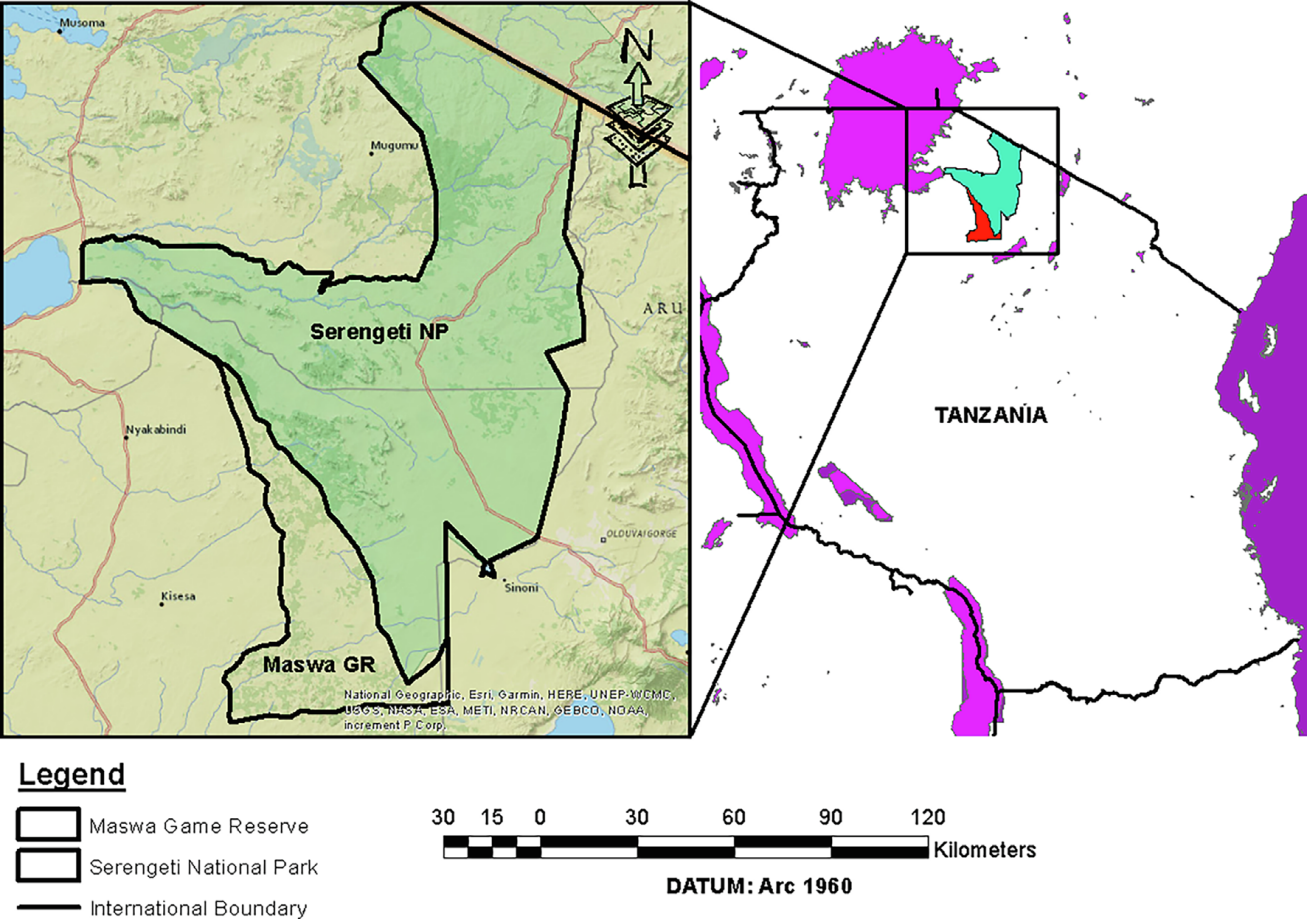
Map of Tanzania illustrating study areas located in the Serengeti ecosystem.

## Discussion

The Serengeti ecosystem is a mega biodiversity hotspot with an enormous number of wildlife species and a key area for understanding and managing disease ecology, including parasite infestation [14, 39, 40]. The presence of third instar larvae in zebra and its rare occurrence in the African lion (*Panthera leo*) [15] reveals a substantial venture to investigate host-parasite dynamics, ecological interactions, and evolutionary adaptations. The infestation of *G*. *intestinalis,* a common obligatory parasite of zebra, aligns with existing knowledge of this parasite in equids, including horses, mules and donkeys [1, 3, 4, 17, 25]. The transmission, variability in prevalence and infection size of *G. intestinalis* are associated with variations in geographical location, fly activity, parasite strain and collective behavioural interactions of the host [17, 20, 30, 41]^.^ The overlapping habitats and various ecological events such as temperature, humidity and precipitation patterns significantly affect the hatching, survival, viability and development rates of larvae, and intensify the risks of infestation in zebra [42]. However, the unusual host infestation may not provide an optimal environment for the parasite development, potentially affecting its lifecycle and pathogenicity of the larvae [18].

This study is the first to report a rare case of *G. intestinalis* in the African lion (*Panthera leo*) from the Serengeti ecosystem. The infection of *G. intestinalis* larvae in the African lion could be due to prey-predator incidences [7, 9] where the lion consumes prey that has developed the third larva stage of *G. intestinalis.* Alternatively, the infestation may result from the deposition of *G. intestinalis* eggs by females onto the lion’s coat. The eggs hatch into the first instar larvae (L1) which are then transferred into the lion’s mouth cavity through licking behaviour [44]. Once in the mouth, the L1 larvae are either ingested or crawl into the mouth cavity, where they moult into the second stage (L2) and eventually move into the stomach [3]. The observed lion’s compromised health may have facilitated the successful moulting and attachment of the L2 and later L3 larvae in the stomach lining, leading to development of the infestation.

The presence of *G. intestinalis* larvae in African lion suggest a possible reason that might justify the adaptive flexibility of Diptera and ectoparasites to exploit non-specific hosts when primary hosts are unavailable [33]. This flexibility demonstrates an evolutionary approach to enhance survival and propagation of parasites in various hosts [33], including lion along the Serengeti ecosystem.

Apart from the occurrence of infection by *G. intestinalis* larvae in Zebra and African lion found in this study, *Gasterophilus* species have been reported in other non-specific hosts, such as humans and dogs [45]. In *Gasterophilus* infestation causing myiasis in humans, the species act as facultative parasites with sporadic incidences in temperate regions, influenced by livestock operations that increase botfly exposure [26]. Poor hygiene practices play a key role in *Gasterophilus* infestation particularly in infants and older age groups [46]. Gastrointestinal myiasis caused by ingestion of contaminated food with eggs or maggots may cause nausea, malaise, vomiting and sometimes cutaneous larval migrans [6, 11]. Conversely, free roaming dogs in areas where botflies are active have a high risk of infestation through ingesting *Gasterophilus* eggs or larvae while licking or grooming themselves after contact with contaminated environments or infested equids and other natural host [45].

We found gastrointestinal wall with anterior spines and mouth hooks of the third larvae on the stomach mucosa. The localization of larvae within the stomach of the African lion and zebras contributes to the broader understanding of the life cycle of *G. intestinalis* and potential physiological implications for the host. The mechanical irritation and inflammation induced by *Gasterophilus* larval attachment can disrupt normal digestive processes, potentially damage stomach tissue, and cause ulceration, chronic gastritis, peritonitis, oesophageal paralysis, squamous cell tumours, and anaemia [10, 35, 37]. Chronic infestations may lead to weight loss, reduced growth, and changes in feeding behaviour [10] that may increase the vulnerability to *G. intestinalis* in zebra and African lion, reflecting the overall impact on the health of the host. In severe cases, particularly with heavy infestations or specific host factors, the cumulative effects may contribute to mortality, especially when secondary complications become systemic [31]. However, their impact in carnivores including African lion in Tanzania is not highly explored.

Wildlife species are natural hosts that have evolved specific immune response to mitigate the effects of infections [36]. However, the lion examined in this study was observed to be weak with shaggy fur and several lesions were found in the mucosal lining that likely contributed to its poor health condition and eventual death. This study proposes comparative research into the immune response of African lion and zebra to provide insights into host-parasite coevolution.

We unveil noteworthy findings in the ecological context and evolutionary significance of the lion and zebra populations in Tanzania. The observed mean nucleotide composition in the *Cox2* gene for both zebra (GC = 0.32, AT = 0.67) and lion (GC = 0.33, AT = 0.66) isolates aligns with the established insect mitochondrial genes, as outlined in earlier studies [28]. The examination of *Cox2* genes within *Gasterophilus* species, sourced exclusively from a singular geographic location, has yielded compelling evidence of notable genetic distinctions and host specificity. For instance, the *Cox2* region was characterised by an observed intraspecies variability of 0.2% in *G. intestinalis* isolated from the African lion and zebra, whilst inter-species variability ranges from 8.5 to 16% on pairwise distance, facilitating precise genetic discrimination of *Gasterophilus* populations and other species used in the study of the phylogenetic relationship.

This study reveals the taxon lineage of the family Gasterophilidae and Oestridae, which were close related compared to the family Tachinidae and Sarcophagidae. These findings provide evidence elucidating host preferences and affirm the paramount importance of molecular taxonomic tools in delineating host-parasite dynamics within this confined geographic region [29]. The unveiled details hold profound implications for targeted ecological management and conservation strategies.

Our current research aligns with and extends these seminal findings, aiming to refine the application of *Cox2* as a molecular marker for *Gasterophilus* identification within the Great Serengeti ecosystem as a unique ecosystem of wildlife species, including African lion and zebra. As we contribute to this evolving body of knowledge, our study not only adds specificity to *Gasterophilus* taxonomy, but also holds broader implications for the fields of wildlife management, evolutionary biology, and host-parasite interactions.

## Conclusion

Our study reported on the occurrence of *G. intestinalis* in Zebra and African lion based on both morphology and molecular studies along the Maswa Game Reserve and Serengeti National Park within the Great Serengeti ecosystem as a new endemic area for the first time. The assessment of parasite diversity at a molecular level contributes crucial data for ecological assessments, aiding in the discernment of ecosystem health and the preservation of biodiversity. The identification of keystone species, such as African lion, and the formulation of sustainable management strategies, guided by molecular characteristics, highlight the ecological relevance of these methodologies. Research on *Gasterophilus* species in most parts of Tanzania is sparse. We addressed the extensive study of *Gasterophilus* species by distinguishing various species available with host-specificity, as a high number of *Gasterophilus* species could infect equids and carnivorous mammals.
